# Golgi Phosphoprotein 73: The Driver of Epithelial-Mesenchymal Transition in Cancer

**DOI:** 10.3389/fonc.2021.783860

**Published:** 2021-12-07

**Authors:** Yiming Liu, Xinyang Hu, Shiyao Liu, Sining Zhou, Zhi Chen, Hongchuan Jin

**Affiliations:** ^1^ Laboratory of Cancer Biology, Key Laboratory of Biotherapy of Zhejiang Province, Sir Run Run Shaw Hospital, Zhejiang University School of Medicine, Hangzhou, China; ^2^ State Key Laboratory for Diagnosis and Treatment of Infectious Diseases, Collaborative Innovation Center for Diagnosis and Treatment of Infectious Disease, The First Affiliated Hospital, Zhejiang University School of Medicine, Hangzhou, China; ^3^ Cancer Center, Zhejiang University, Hangzhou, China

**Keywords:** GP73, cancer biomarker, epithelial mesenchymal transition, cancer metastasis, protein trafficking

## Abstract

Golgi phosphoprotein 73 (GP73, also termed as GOLM1 or GOLPH2) is a glycosylated protein residing on *cis*-Golgi cisternae and highly expressed in various types of cancer tissues. Since GP73 is a secretory protein and detectable in serum derived from cancer patients, it has been regarded as a novel serum biomarker for the diagnosis of different cancers, especially hepatocellular carcinoma (HCC). However, the functional roles of GP73 in cancer development are still poorly understood. In recent years, it has been discovered that GP73 acts as a multifunctional protein-facilitating cancer progression, and strikingly, it has been identified as a leading factor promoting epithelial-mesenchymal transition (EMT) of cancer cells and causing cancer metastasis. In this review, we have overviewed the latest findings of the functional roles of GP73 in elevating cancer progression, especially in facilitating EMT and cancer metastasis through modulating expression, transactivation, and trafficking of EMT-related proteins. In addition, unsolved research fields of GP73 have been lightened, which might be helpful to elucidate the regulatory mechanisms of GP73 on EMT and provide potential approaches in therapeutics against cancer metastasis.

## Introduction

In the past decades, cancer has been ranked as the primary cause of death and the largest health problem worldwide (1). The incidence and mortality of cancer are rapidly rising because of aging, growth of the population, environmental pollution, as well as other social problems (1–3). Metastasis and recurrence are the main causes of cancer-related deaths (4). A hypoxic tumor microenvironment is created since a large amount of oxygen is consumed in the metabolism of cancer cells, which challenges the survival of cancer cells. As a result, cancer cells have to shift to places with high oxygen concentrations to promise cell metabolism and resist cell death, the process of which is named cancer metastasis (5). Cancer progression towards metastasis is often depicted as a multistage process and cancer cells achieve metastasis through epithelial-mesenchymal transition (EMT) during the process (6–8). When cancer cells demonstrate a shift towards the mesenchymal state, expression, and modifications of EMT-related molecules are changed, which shape cells into spindle, then, migratory and invasive behaviors of cancer cells are facilitated (9). Some EMT-like cancer cells named as circulating tumor cells (CTCs) invade into blood vessels and migrate with the bloodstream. CTCs can be clustered to evade immune defense and enhance survival of cancer cells in the blood, moreover, high expression of CD44, the cancer-specific surface antigen, facilitates heterotypic adhesion of CTCs, which promises the distant metastasis of CTCs and strives for more nutrients (10, 11).

EMT-associated transcription factors (TFs), such as ZEB1, SNAIL1, and TWIST1 transactivate EMT factors associated with cell adhesion, migration, and invasion (12–14). The repression of such transactivation was proposed as a rational strategy to reverse EMT. However, these well-known EMT-associated TFs are differentially expressed in various cancer types. Therefore, it is valuable to discover a protein regulating most of EMT-related factors, thus allowing a specific small-molecule inhibitor to potentially target EMT of cancer cells.

## Characteristics and Structure of GP73

**Figure 1:**
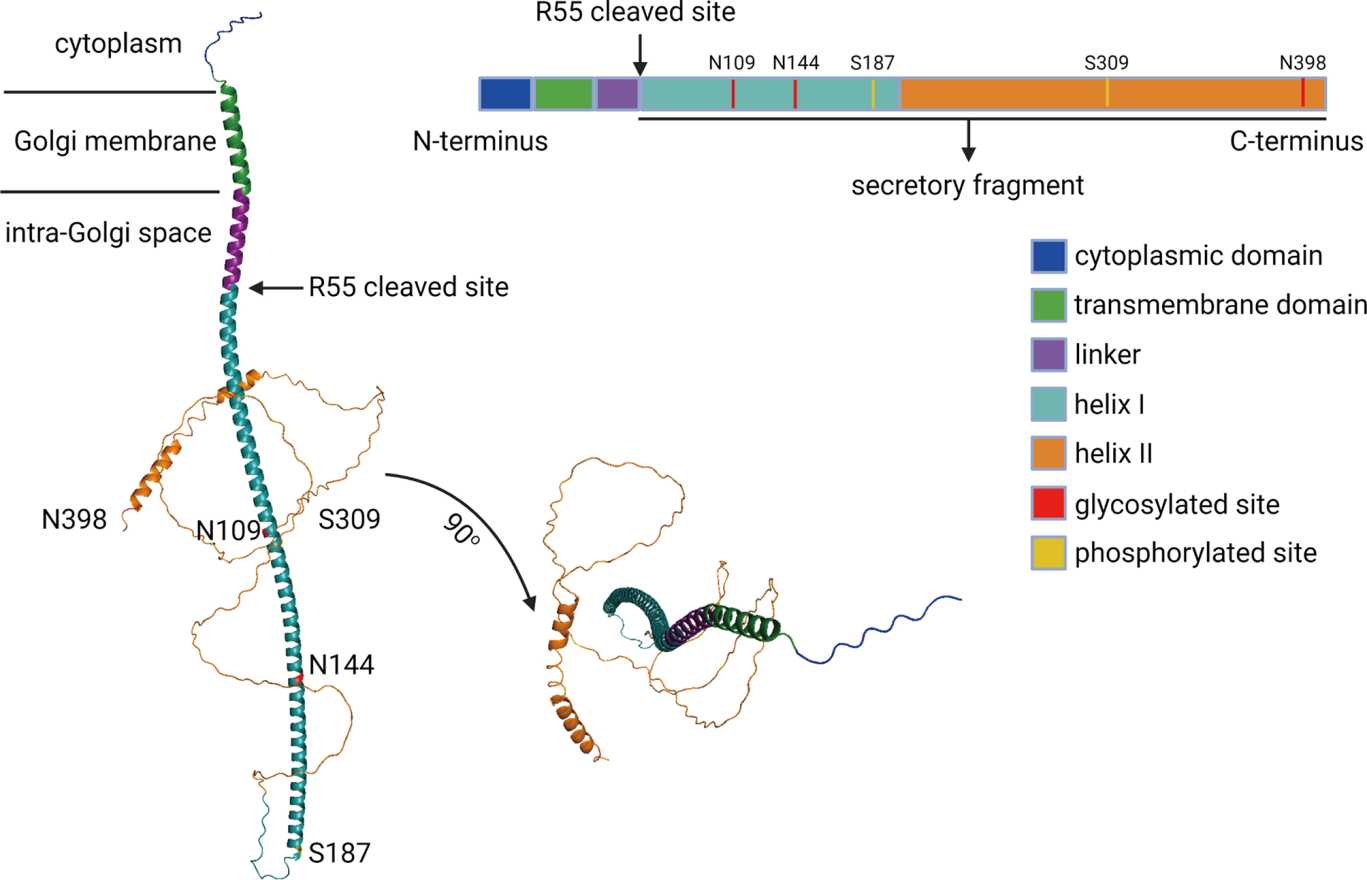
The schematic diagram of GP73. The functional domains, phosphorylated sites, and glycosylated sites of full-length GP73 has been showed. The predicted molecular structure of GP73 was obtained from AlphaFold Protein Structure Database (https://alphafold.ebi.ac.uk).

In 2000, a novel protein named Golgi phosphoprotein 73 (GP73, also termed as GOLM1 or GOLPH2) was identified and isolated from the liver of a patient who suffered from adult giant-cell hepatitis (GCH), a rare form of hepatitis with presumed viral etiology (15). GP73 is encoded by *GOLM1*, and the open reading frame comprises two regions encoding products containing 392 and 401 amino acids (aa) (16). GP73 resides in *cis-*Golgi cisternae, and it contains a transmembrane domain (TMD) at the N-terminal region (13–35aa) and two α-helixes at the C-terminal region (56–205 and 206–401aa) (16, 17). The cytoplasmic region of GP73 is formed by 1–12aa and, remarkably, GP73 interacts with its substrates *via* this domain and involves in the vesicular trafficking of these proteins (17–19). Also, three N-linked glycosylated sites (N109, N144, and N398) and two phosphorylated sites (S187 and S309) have been detected using liquid chromatography and high-throughput mass spectrometry (LC-MS/MS), but the exact functions of these modifications remain poorly understood (**Figure 1**) **(** 20–23). Intracellular vesicles engage in the trafficking process of GP73 from the Golgi apparatus to cell surface, and the secretion of GP73 from the cell surface to extracellular spaces is exosome dependent (18). Furin has been identified as a proteinase to exclusively cleave GP73 at R55 on the intracellular side of the cell surface, which permits the remaining part of GP73 (56–401aa) covered by exosomes and secreted into extracellular spaces *via* exosome-dependent secretion (24). Therefore, 56–401aa residue of GP73 is detectable in extracellular spaces and potentially used as a serum biomarker for the diagnosis of cancers (25).

## GP73 Serves as a Biomarker in Cancer Diagnostics

In 2005, a study based on glycoproteomics screened serum glycoproteins and identified serum GP73 as a factor positively correlated with human hepatocellular carcinoma (HCC), which suggested that GP73 serves as a potential serum biomarker for HCC diagnosis (26). Following studies indicate that intracellular GP73 correlates positively with extracellular GP73, and both of them could be potentially used as biomarkers for diagnosis of HCC (23, 24, 27, 28). Notably, GP73 has been indicated to be highly expressed in pathological tissues and serum derived from early cancer patients, which manifests higher diagnostic sensitivity and specificity than classic HCC biomarker alpha-fetoprotein (AFP) (29–31). Thus, GP73 has been used as a novel serum biomarker for clinical diagnostics of HCC. Two follow-up studies have uncovered that GP73 is highly expressed in prostate cancer tissues, which indicate that GP73 may not be an HCC-specific biomarker but potentially applicable for diagnosis of pan-cancers (32, 33). Further studies examined the level of GP73 in different types of cancers, and the results reveal that GP73 is not only an HCC-specific biomarker but also serves as a suitable biomarker for diagnosis of other malignant tumors (**Table 1**). Similar to HCC, GP73 is also detectable in most other types of early cancers, which suggests that GP73, as a comprehensive and sensitive biomarker, is expected to be applied in clinical diagnostics of different types of cancers.

**Table 1:** GP73 is highly expressed in pathological tissues and serum derived from cancer patients.

Functional system	Tumor type	Sample type	Clinical outcome	Ref.
Digestive system	HCC	Tissues and serum	Poor	(17, 25, 34–37)
	Gastric cancer	Tissues and serum	Poor	(38, 39)
	Pancreatic cancer	Tissues	Poor	(40, 41)
	ESCC	Tissues	Poor	(42)
	OSCC	Tissues	Poor	(43)
Respiratory system	NSCLC	Tissues and serum	Poor	(44–47)
Integumentary system	Cutaneous melanoma	Tissues	Poor	(48)
Nervous system	Cerebroma	Tissues	Not mentioned	(49, 50)
Urinary system	Prostate cancer	Tissues and urines	Poor	(32, 33, 51–53)
	Renal cell cancer	Tissues	Poor	(54)
	Bladder cancer	Tissues	Poor	(55)
Reproductive system	Seminoma	Tissues	Not mentioned	(56)
	Cervical cancer	Tissues and serum	Poor	(57)

ESCC, esophageal squamous cell carcinoma; OSCC, oral squamous cell carcinoma.

## Transactivation and Expression of GP73 in Cancer Cells

Years after GP73 was identified, the discoverer of GP73 measured the protein level of GP73 in pathological tissues derived from patients with different liver diseases and found that GP73 was highly expressed in patients suffering from acute hepatitis of various etiologies, autoimmune hepatitis, chronic hepatitis C virus (HCV) infection, and alcoholic liver diseases (58). Additional studies have indicated that GP73 is highly expressed in hepatitis B virus (HBV)-infected liver tissues compared with non-HBV-infected liver tissues, implying that viral infection might upregulate expression of GP73 (59–61). Pathogen-associated molecular patterns can be recognized by pattern recognition receptors during the process of viral infection, which leads to the activation and secretion of interferons (IFNs) (62). Therefore, it is supposed that virus might activate GP73 expression *via* stimulating the expression and secretion of IFNs. Indeed, a recent study reveals that IFN-β activates GP73 expression and represses innate immune response in viral-infected HCC cells through facilitating the degradation of mitochondrial antivirus signaling protein (MAVS)/TNF receptor-associated factor 6 (TRAF6) and attenuating *IFN-β* promoter (63). However, some other studies indicate that serum GP73 might not be a suitable diagnostic marker for HCC because HBV infection rather than tumorigenesis facilitates GP73 expression (64, 65). Nevertheless, following studies discovered that GP73 is also highly expressed in carcinomas without viral infection, such as nonsmall-cell lung cancer (NSCLC), cutaneous melanoma, cerebroma, prostate cancer, renal cell cancer, and bladder cancer, which suggest that biogenesis of GP73 is regulated by multiple factors and the mechanism is complex (**Table 2**).

**Table 2:** Transactivation and expression of GP73 in cancer cells.

Type of regulation	Effect	Type of tissue	Regulation	Ref.
Micro-RNA	Reduction of miR-27b	HCC; prostate cancer	Up	(66, 67)
	Reduction of miR-128-3p	Pancreatic cancer	Up	(68)
	Reduction of miR-143	Cervical cancer; prostate cancer	Up	(69, 70)
	Reduction of miR-145	Pan-cancer	Up	(70, 71)
	Reduction of miR-200a	Lung adenocarcinoma	Up	(72)
	Reduction of miR-212-3p	Breast cancer	Up	(73)
	Reduction of miR-382	HCC	Up	(74)
	Reduction of miR-384	Glioma	Up	(75)
	Reduction of miR-493-5p	HCC	Up	(76)
	Reduction of miR-653	HCC	Up	(77)
	Reduction of miR-3935	Prostate cancer	Up	(78)
Cell signaling	IFN-β activation	Chronic HCV-infected HCC	Up	(63)
	mTORC1 activation	HCC	Up	(34)
Transactivation	ETS-1	HCC	Up	(79)
	c-Myc	HCC	Up	(19)
Infection	HBV infection	Chronic HBV-infected HCC	Up	(80–82)
	HCV infection	Chronic HCV-infected HCC	Up	(63, 83)
	Adenovirus infection	HCC cell lines	Up	(84)
	Bacteria and fungi infection	Lymphocytes	Up	(85)

With the rise of researches about micro-RNA (miRNA) in recent years, some studies manifest that the levels of multiple miRNAs targeting the 3′-untranslated region (3′-UTR) of *GOLM1* are attenuated in cancer cells, but the regulatory mechanism is still unclear (**Table 2**). As is well known, miRNAs are not the dominant factors regulating protein expression, it is significant to explain how GP73 is transactivated in viral-infected cells and cancer cells (86–88).

**Figure 2:**
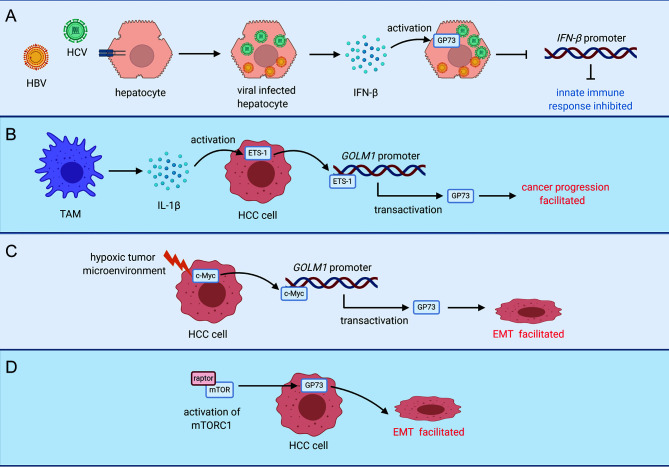
Activation modes of GP73 in viral infected cells or cancer cells. **(A)** INF-β secreted by viral-infected hepatocytes activates GP73 in target cells and inhibits innate immune response. **(B)** TAM-secreted IL-1β upregulates ETS-1 in cancer cells and ETS-1 transactivates GP73 to facilitate cancer progression. **(C)** Upregulation of c-Myc in a mildly hypoxic tumor microenvironment transactivates GP73 and facilitates EMT of cancer cells. **(D)** GP73 is upregulated by mTORC1, and EMT of cancer cells is facilitated.

As extracellular stimulations such as epithelial growth factor (EGF), tumor-associated macrophages (TAMs), and immune suppression-related cytokines in tumor microenvironment facilitate cancer progression, it is believed that extracellular factors in tumor microenvironment might play important roles in facilitating GP73 expression (89). It has been discovered that TAM-originated interleukin-1β (IL-1β) can activate the expression of ETS-1, the well-known oncogenic TF, which interacts with the promoter of *GOLM1* and promotes its transcription (79). Similarly, a recent study in our group has uncovered that hypoxia upregulates oncogenic protein c-Myc and transactivates GP73 in a mildly hypoxic tumor microenvironment, which suggests that GP73 might be activated to play critical roles against adverse circumstances and promote the survival of cancer cells (**Figure 2**) **(** 19).

It is known that the mammalian target of rapamycin complex 1 (mTORC1) is involved in physiological processes including protein synthesis, cell metabolism, tumor proliferation, and autophagy; however, its functional roles in cancer cells are still poorly understood since the regulatory mechanisms are complex (90–92). An early study has reported that mTORC1 upregulates GP73 in HCC cells and promotes cell proliferation (34). It is well-known that activation of mTORC1 facilitates phosphorylation of p70-S6 kinase (S6K) and eukaryotic translation initiation factor 4E-binding protein 1 (4EBP1), two factors that engage in protein synthesis, and accelerate HCC proliferation (93). This study, however, has indicated that GP73 directly upregulates S6K, then promotes protein synthesis and cell proliferation. Therefore, GP73, as the downstream protein of mTORC1, plays synergistic roles with mTORC1 in facilitating carcinogenesis. Additionally, RNA sequencing in this study has revealed that knockdown of GP73 reduces the levels of matrix metalloproteinase-7 (MMP-7) and CD44, two factors involved in cell invasion, heterotypic adhesion, and HCC metastasis; however, the regulatory mechanisms are unclear (94–96).

The results above have elucidated how GP73 is activated in cancer cells; furthermore, it is proved that GP73 takes important effects on cancer metastasis as well as cancer proliferation and promotes cancer progression.

## GP73 Promotes EMT of Cancer Cells

It has been mentioned above that GP73 facilitates cancer metastasis as well as proliferation, and GP73 upregulates MMP-7 and CD44, the factors highly expressed in metastatic cancer cells. Therefore, it is deemed that GP73 might promote EMT of cancer cells through upregulating the levels of EMT-related proteins.

Clinical studies have also demonstrated that GP73 is highly expressed in cancer tissues with infiltration (**Table 3**). However, since it is difficult to obtain metastatic tissues after cancer recurrence, no study has reported the expression of GP73 in distant metastatic tissues.

**Table 3:** GP73 expression and its correlation with cancer infiltration.

Tumor types	Sample types	Patients	pTNM (Ⅰ, Ⅱ/Ⅲ, Ⅳ)	GP73 high (50%) (%, Ⅰ, Ⅱ/Ⅲ, Ⅳ)	Correlation	Clinical outcome	Ref.
Bladder cancer	Tissues	*n* = 102	65/37	43.08/94.59	Positive	Poor	(55)
HCC	Tissues	*n* = 80	41/39	63.41/84.62	Positive	Poor	(35)
Pancreatic cancer	Tissues	*n* = 120	80/40	57.50/95.00	Positive	Poor	(40)
Colorectal cancer	Tissues	*n* = 341	203/138	29.56/42.03	Positive	Poor	(97)
HCC	Tissues	*n* = 239	236/3	46.19/66.67	Positive	Poor	(98)
NSCLC	Tissues	*n* = 37	26/11	65.38/63.64	Positive	Poor	(46)
Gastric cancer	Tissues	*n* = 385	141/244	48.23/64.75	Positive	Poor	(38)
HCC	Tissues	*n* = 91	83/8	48.19/75.00	Positive	Poor	(17)
HCC	Tissues	*n* = 75	15/60	20.00/85.00	Positive	Poor	(99)

Whatever, in recent years, an increasing number of studies have illustrated the functional roles of GP73 in cancer metastasis. In a pioneering study, with the help of laser-capture tissue microdissection and genome-wide cDNA arrays technologies, *GOLM1* was identified as a leading gene significantly upregulated in tumor tissues from HCC patients with extrahepatic metastases (EHMH) but not in tissues from metastasis-free HCC (MFH) patients, which suggests that GP73 is a critical factor modulating cancer metastasis (17). Since metastasis is the main cause of cancer-related death and EMT is the essential condition of metastasis, it is clinically significant to investigate the regulatory mechanisms of GP73 on cancer metastasis (100). Following studies have focused on the mechanisms of how GP73 facilitates cancer metastasis, and they have discovered that highly expressed GP73 upregulates the levels of N-cadherin, vimentin, and MMP-13 in HCC cells, which prove that GP73 surely serves as a multifunctional factor modulating the expression of EMT-related proteins (**Table 4**). Also, GP73 negatively regulates the expression of E-cadherin, the well-known adhesion factor, and promotes EMT through attenuating cell adhesion (103, 108). One of these studies has demonstrated that GP73 upregulates c-AMP element response binding protein (CREB), a common TF highly expressed in cancer cells, and transactivated MMP-13; however, the mechanism is not totally elucidated and no other EMT-associated TFs have been discovered to be regulated by GP73 (104, 109, 110).

**Table 4:** GP73 regulates expression and trafficking of EMT-related factors and facilitates cancer metastasis.

Type of regulation	EMT factor	Type of tissue	Regulation	Ref.
Glycosylation at Asn 144	Not Applicable	HCC	Inhibits EMT	(101)
Regulates the levels of EMT-related factors	N-cadherin	HCC; pancreatic cancer; bladder cancer	Up	(40, 55, 102)
	E-cadherin	HCC; pancreatic cancer; bladder cancer	Down	(35, 40, 55, 99, 103)
	Vimentin	HCC; bladder cancer	Up	(35, 40, 55, 99, 103)
	CD44	HCC; cerebroma	Up	(34, 49)
	MMP-13	HCC; cervical cancer; NSCLC	Up	(46, 57, 104)
	MMP-7	HCC	Up	(34)
Involves in the trafficking of EMT-related factors	EGFR	HCC	Translocation	(17)
	MMP-2	HCC	Translocation	(18)
	MMP-7	HCC	Translocation	(19)
	AFP	HCC	Translocation	(105)
Extracellular GP73	sGP73	Esophageal cancer	Facilitates EMT	(106)
	GP73-exo	HCC	Facilitates EMT	(107)

sGP73, secretory GP73; GP73-exo, exosomal GP73.

On the other hand, since GP73 is a highly glycosylated and phosphorylated protein, it is supposed that specific modified sites of GP73 might impact the process of EMT. LC-MS/MS analysis has discovered that GP73 is N-glycosylated at Asn109, Asn144, and Asn398 (101). Following analyses have demonstrated that removal of N-linked glycosylation of GP73 at Asn144 enhances metastasis of HCC cells, which proves that modified sites of GP73 impact its functions in facilitating EMT. It is believed that other phosphorylated and glycosylated sites might also take effect on EMT, which is worth further exploring.

## GP73 Acts as a Transporter of EMT-Related Proteins

As described, GP73 facilitates EMT of cancer cells through regulating the expressions of EMT-related proteins, but the mechanisms are still poorly understood. For solving these puzzles, GP73-interacted proteins were identified using coimmunoprecipitation combined with LC-MS/MS, and epithelial growth factor receptor (EGFR) was identified as a critical GP73-interacted factor in HCC cells, which interacts with GP73 *via* the cytoplasmic domain of GP73 (17, 111, 112). Since GP73 is a transmembrane protein and the cytoplasmic domain resides on the outside of the membrane of *cis-*Golgi cisternae and intracellular vesicles, it is suggested that EGFR is translocated onto the cell surface and exerts its biological functions through GP73-dependent vesicular trafficking. Following fluorescent protein-based live-cell imaging and functional experiments have proved the hypothesis. The study above has indicated that GP73 acts as a transporter facilitating the trafficking and translocation of EMT-related proteins, promising EMT of cancer cells promoted.

Similarly, in our early studies, it was observed that knockdown of GP73 induced the accumulation of intracellular matrix metalloproteinase-2 (MMP-2) and MMP-7 but attenuated the levels of extracellular MMP-2 and MMP-7, which suggests that knockdown of GP73 might block the trafficking and secretion of matrix metalloproteinases (MMPs) (18, 19). Further pieces of evidence prove that, as well as GP73/EGFR interaction, MMP-2 and MMP-7 interact with the cytoplasmic domain of GP73, and GP73 is involved in their translocation from cytosol to extracellular spaces through GP73-mediated vesicular trafficking. These findings have manifested that the trafficking of MMPs is GP73 dependent. Therefore, GP73 has been deemed as a transporter for trafficking of EMT-associated factors and facilitating EMT of cancer cells.

Also, it has been revealed that GP73 interacts with Rab11, a lysosome-dependent degradation-related protein residing on the membrane of intracellular vesicles, and mediates lysosome-dependent degradation of EGFR (17, 113). When weak signal is activated, the GP73-Rab11 complex mediates the trafficking of EGFR from *cis-*Golgi cisternae to lysosome and promotes the degradation of EGFR. Oppositely, GP73 facilitates the polarized delivery of EGFR from *cis-*Golgi to the plasma membrane when EGFR signaling pathway is activated, which enhances the activation of EGFR signaling pathway. The findings show that GP73 is a switch-modulating metastasis, metabolism, and dormancy of HCC cells through regulating the translocation of growth factor receptors. It has also indicated that GP73 not only modulates the trafficking of EMT-related proteins from cytosol to cell surface or extracellular spaces but also involves in protein recycling and energy saving.

Moreover, an updated study indicated that GP73 directly interacted with AFP and facilitated its secretion, which led to EMT of recipient cells of AFP and promoted immune escape of cancer cells (105).

Taken together, GP73, as a *cis-*Golgi cisternae-resided protein, exerts its functional roles in the trafficking and recycling of EMT-related factors and promotes EMT of cancer cells.

## Extracellular GP73 Facilitates EMT of Cancer Cells

As described, intracellular GP73 can be cleaved at the trans-Golgi network (TGN) due to saturation or mini-stack formation, or cleaved by furin proteinase on the intracellular side of cell surface, then released into extracellular spaces *via* exosomes (18, 24, 106). The studies have elucidated how GP73 is cleaved and secreted into extracellular spaces; however, the functional roles of extracellular GP73 remain poorly understood. A previous study has indicated that overexpression of GP73 1-55aa-deleted truncated mutant facilitates cell invasion (104). Since exosomal GP73 shares an identical sequence and structure with GP73 1-55aa-deleted truncated mutant, it is supposed that exosomal GP73 might facilitate metastasis of neighboring cancer cells while it is captured by recipient cells. A recent study has discovered that mTOR upregulates GP73 through reducing the level of miR-145, the miRNA targeting 3′UTR of *GOLM1*, and exosomal GP73 facilitated proliferation and invasion of neighboring cancer cells by upregulating glycogen synthase kinase-3β (GSK-3β) and MMPs (107). Though exosomal GP73 upregulates proliferation and cell invasion-related proteins of recipient cells, it has not elucidated how it activates the expressions of these target factors. Therefore, the molecular mechanisms need further investigations.

The studies above have indicated that cancer cell-originated exosomal GP73 acts as a messenger that functionally activates growth and EMT of recipient cells, which suggests that exosomal GP73 plays vital roles in cell-to-cell interactions in cancer microenvironment.

## Conclusion and prospective

**Figure 3:**
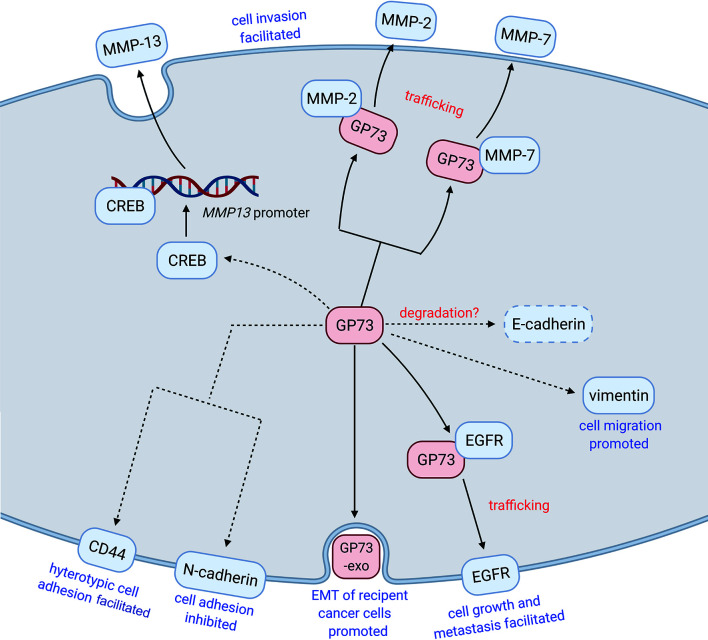
GP73 promotes EMT of cancer cells through different pathways. GP73, as a multifunctional protein, facilitates EMT of cancer cells through regulating the levels, promoting the transactivation, and accelerating the trafficking of EMT-related proteins. Exosomal GP73 also plays functional roles in promoting EMT of recipient cancer cells.

GP73 plays functional roles in facilitating EMT of cancer cells through multiple pathways, which proves that GP73 goes beyond a tumor biomarker for cancer diagnosis (**Figure 3**). As previously reported, *GOLM1* has been identified as a leading gene associated with cancer metastasis; it is supposed that GP73 serves as a potential drug target in therapeutics of metastatic cancers (17). Fortunately, as a tumor biomarker, GP73 expresses little in normal tissues, and previous studies have proved that GP73 deletion impacts little on the physiological activities of mice (25, 34). Therefore, it is significant to explore small-molecule inhibitors targeting GP73 for potential therapeutics against cancer metastasis. As reported that tunicamycin, the drug inhibiting N-linked glycosylation of proteins, prevents the glycosylation of GP73 and attenuates its functions in facilitating HCC metastasis, it is potentially utilized in cancer therapy (34, 114). However, since it is a comprehensive inhibitor targeting almost all glycosylated proteins and inducing high cytotoxicity to normal cells, it is not suitable for GP73-targeted clinical therapeutics. Therefore, it is interesting and important to explore novel GP73-specific inhibitors for therapeutics against cancer metastasis.

Since GP73 facilitates EMT of cancer cells through regulating expression, trafficking, and secretion of EMT-related proteins, it is supposed that GP73 acts as a vital factor exerting a wide range of physiological functions in cancer cells and the functional roles of GP73 in cancer microenvironment are far more beyond our recognition. Herein, five concerning issues about GP73 are highlighted and further studies might be helpful to explain the regulatory mechanisms and confirm the diagnostic ranges of GP73.

Above all, as described, GP73 is involved in the trafficking of EGFR, MMP-2, and MMP-7. It is considered a transporter assisting the trafficking of EMT-related factors. Therefore, GP73 might facilitate proliferation and metastasis of cancer cells by promoting the trafficking of carcinogenesis-associated cell-surface receptors or secretory proteins. It is believed that more substrates might facilitate EMT of cancer cells through GP73-mediated trafficking.

Secondly, GP73 also plays functional roles in promoting proteasome-dependent degradation of target proteins, such as MAVS and TRAF6 (63). Therefore, GP73 is not only a transporter facilitating the trafficking of cell surface and secretory proteins but also a recycler promoting degradation of intracellular proteins. As shown that overexpression of GP73 reduced the level of E-cadherin, it is worth investigating whether GP73 is involved in proteasome-dependent degradation of E-cadherin (35). In addition, since it has been revealed that GP73 is also involved in the lysosome-dependent degradation of target proteins, it is also interesting and meaningful to discover its substrates in lysosome-dependent degradation (17).

Thirdly, as mentioned above, exosomal GP73 facilitates cell proliferation and metastasis through activating GSK-3β and MMP-related signaling pathways (107). However, it is unclear how GP73 activates these signaling pathways. Therefore, LC-MS/MS and RNA sequencing are essential here to identify the exosomal GP73-interacted proteins and mechanically explain how it facilitates cell growth and EMT.

Fourthly, the pieces of evidence above have indicated that cancer cell-originated exosomal GP73 facilitates growth and EMT of neighboring cells, which implies that it might exert important functions in tumor microenvironment. An early study has described that exosomal GP73 induces endoplasmic reticulum stress of macrophages, which stimulates the secretion of cytokines and chemokines involved in the formation of TAMs (115). Also, two recent studies have reported that GP73 upregulates programmed cell death ligand-1 (PD-L1) and facilitates immune escape of HCC cells through activating EGFR signaling pathway, which prove that, similar to the former study, GP73 also plays key roles in immunomicroenvironment (98, 116). On the contrary, another latest study has indicated that GP73 maintains the intestinal epithelial barrier and suppresses carcinogenesis of colorectal carcinoma (CRC) through restraining protumorigenic inflammation (117). Thus, GP73 not only regulates cell growth and EMT but also involves in immunoregulation and indirectly modulates cancer progression, which deserves further investigation.

Fifthly, since HBV infection upregulates the expression of GP73, its diagnostic values in HCC and other liver diseases are challenged (65). More pathological samples derived from HBV or non-HBV-infected HCC patients are expected to be analyzed to clarify its range of application in diagnostics.

Lastly, since it has been reported that knockdown of GP73 could inhibit cancer proliferation and metastasis *in vitro* and *in vivo*, it is deemed that GP73 might serve as a potential drug target (17, 19, 34, 115). Therefore, it is meaningful to explore small molecule inhibitors targeting intracellular and extracellular GP73 and assess their application values in cancer therapeutics.

**Figure 4:**
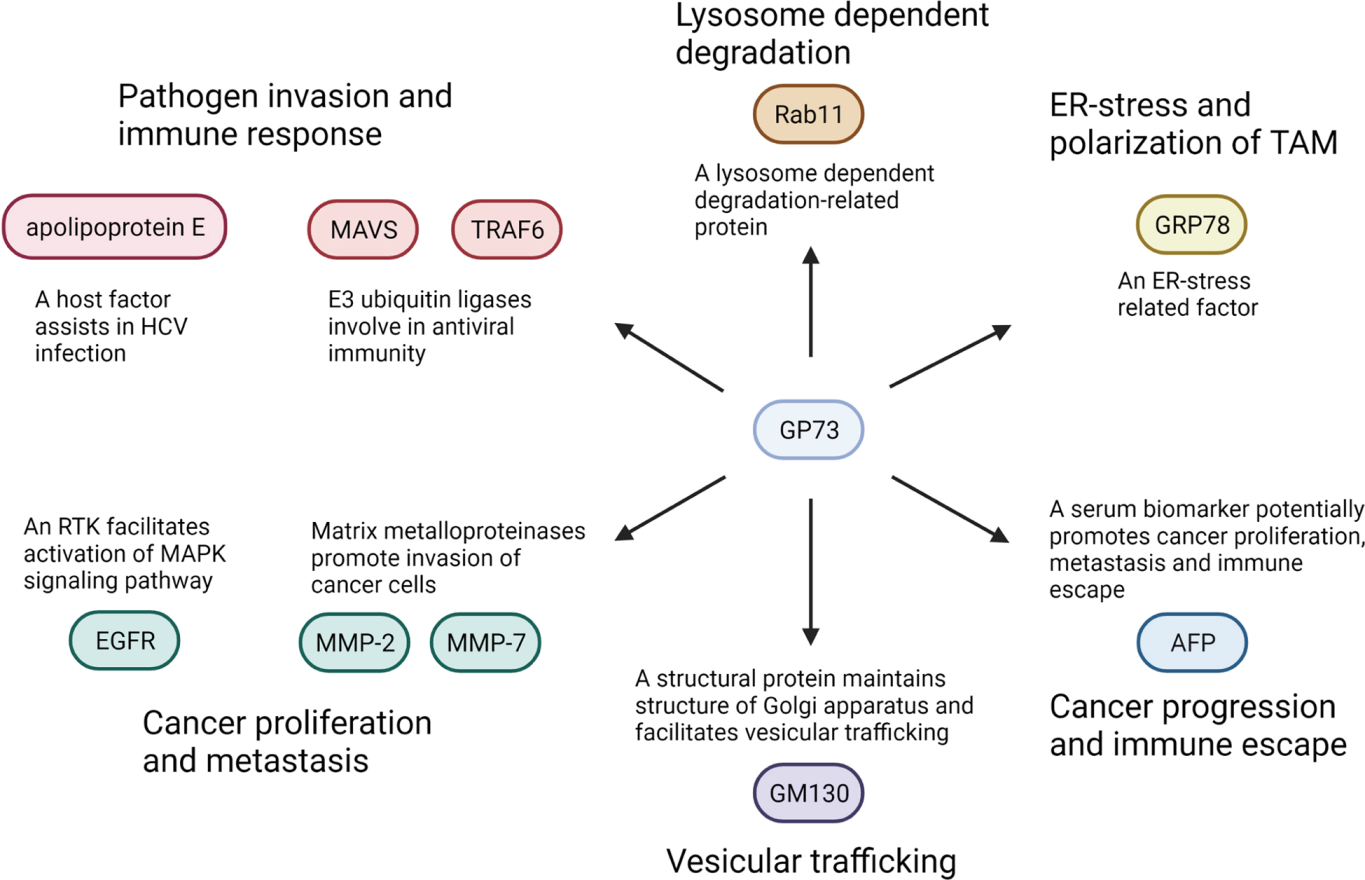
GP73-interacted proteins and their functions in physiological and biomedical processes. GP73 interacts with indicated proteins and facilitates cancer progression or pathogen invasion through various pathways.

Herein, the GP73-interacted proteins and functions have been summarized, which might help readers to comprehend its actions in physiological and biochemical processes (**Figure 4**). Though it has been known that GP73 is a critical factor facilitating EMT of cancer cells, the functions of which are still beyond our recognition. The highlighted points above might illuminate us to gradually uncover the physiological functions of GP73 in cancer cells, which might be helpful in the diagnosis and treatment of cancer metastasis.

## Author Contributions

YL assigned the outlines of the manuscript and wrote the manuscript. XH collected relevant references and produced tables. SL analyzed clinical data in **Table 3**. SZ drew the figures. ZC and HJ revised the manuscripts. All authors contributed to the article and approved the submitted version.

## Funding

This work was supported by the Natural Science Foundation of Zhejiang province (grant numbers LR19H160003), the Natural Science Foundation of Zhejiang province (grant numbers LQ21H160029), and the Medical Science and Health Technology Project of Zhejiang province (grant numbers 2020RC067).

## Conflict of Interest

The authors declare that the research was conducted in the absence of any commercial or financial relationships that could be construed as a potential conflict of interest.

## Publisher’s Note

All claims expressed in this article are solely those of the authors and do not necessarily represent those of their affiliated organizations, or those of the publisher, the editors and the reviewers. Any product that may be evaluated in this article, or claim that may be made by its manufacturer, is not guaranteed or endorsed by the publisher.

## Abbreviations

GP73, Golgi phosphoprotein 73; GOLM1, Golgi membrane protein 1; GOLPH2, Golgi phosphoprotein 2; HCC, hepatocellular carcinoma; EMT, epithelial mesenchymal transition; CTCs, circulating tumor cells; TFs, transcription factors; GCH, giant-cell hepatitis; aa, amino acids; TMD, transmembrane domain; LC-MS/MS, liquid chromatography and high-throughput mass spectrometry; AFP, alpha-fetoprotein; HCV, hepatitis C virus; HBV, hepatitis B virus; IFNs, interferons; NSCLC, nonsmall-cell lung cancer; miRNA, micro-RNA; 3’ UTR, 3′-untranslated region; EGF, epithelial growth factor; TAMs, tumor-associated macrophages; IL-1β, interleukin-1β; mTORC1, mammalian target of rapamycin complex 1; S6K, p70-S6 kinase; 4EBP1, eukaryotic translation initiation factor 4E-binding protein 1; MMP-7, matrix metalloproteinase-7; EHMH, extrahepatic metastases; MFH, metastasis-free HCC; CREB, c-AMP element response binding protein; EGFR, epithelial growth factor receptor; MMP-2, matrix metalloproteinase-2; MMPs, matrix metalloproteinases; TGN, trans-Golgi-network; GSK-3β, glycogen synthase kinase-3β; MAVS, mitochondrial antivirus signaling protein; TRAF6, TNF receptor-associated factor 6; PD-L1, programmed cell death ligand-1; CRC, colorectal carcinoma; ESCC, esophageal squamous cell carcinoma; OSCC, oral squamous cell carcinoma; sGP73, secretory GP73.
